# The Concept of Oppression and Occupational Therapy: A Critical Interpretive Synthesis

**DOI:** 10.1177/00084174211051168

**Published:** 2021-11-02

**Authors:** Elizabeth A. Pooley, Brenda L. Beagan

**Keywords:** Critical reflexivity, Equity, Occupations, Social justice, Social structures, Équité, justice sociale, occupations, réflexivité critique, structures sociales

## Abstract

**Background.** Occupational therapy and occupational science literature include growing attention to issues of justice, marginalization, and rights. In contrast, the concept of oppression has scarcely been employed. **Purpose.** This paper investigates how adding the concept of oppression may enhance occupational therapy approaches to injustice, prioritizing a focus on structural causes, and facilitating conscientious action. **Method.** A critical interpretive synthesis explored insights from authors who name oppressions in occupational therapy and occupational science literature. In total, a sample of 28 papers addressing oppression, ableism, ageism, classism, colonialism, heterosexism, racism, and/or sexism was selected for inclusion. **Findings.** Four themes were identified: oppression and everyday doing; effects of structures and power; responding and resisting; and oppression within occupational therapy. **Implications.** Incorporating oppression within the plurality of social discourse may help occupational therapists to avoid individualistic explanations, attend to relationships between social structures and constrained occupations, frame intersectional analysis, and engage in praxis.

## Abrégé

**Description.** La littérature sur l'ergothérapie et sur la science de l’occupation accorde une attention croissante aux questions de justice, de marginalisation et de droits. En revanche, le concept d'oppression n'a guère été employé. **But.** Cet article étudie comment l'ajout du concept d'oppression pourrait améliorer les approches ergothérapiques de l'injustice, en mettant l'accent sur les causes structurelles et en facilitant l'action consciencieuse. **Méthodologie.** Une synthèse critique et interprétative a exploré les idées des auteurs qui nomment les oppressions dans la littérature sur l’ergothérapie et la science de l’occupation. Au total, un échantillon de 28 articles traitant de l'oppression, du capacitisme, de l'âgisme, du classisme, du colonialisme, de l'hétérosexisme, du racisme et/ou du sexisme a été sélectionné pour être inclus. **Résultats.** Quatre thèmes ont été identifiés : l'oppression et l'action quotidienne; les effets des structures et du pouvoir; la réponse et la résistance; l'oppression au sein de l'ergothérapie. **Conséquences.** L'incorporation de l'oppression dans la pluralité du discours social pourrait aider les ergothérapeutes à éviter les explications individualistes, à s’intéresser aux relations entre les structures sociales et les occupations contraignantes, à encadrer l'analyse intersectionnelle et à s’engager dans la praxis.

## Introduction

Occupational therapists and scholars have interrogated the social conditions that limit occupation (Hammell, 2020; Malfitano et al., 2016). The concept of occupational justice arose from a need to attend to occupations that are “barred, confined, restricted, segregated, prohibited, underdeveloped, disrupted, alienated, marginalized, exploited, excluded or otherwise restricted” (Townsend & Wilcock, 2004, p. 77). Yet, despite sustained attention to justice and injustice, the concept of “oppression” has been largely unnamed by occupational therapy authors. It has been argued that “ethics, moral concepts or codes intended for social professionals that do not foreground such a substantial concept of oppression are objectionable” (Clifford, 2016, p. 16). Indeed, there have been recent international calls for occupational therapy to challenge oppression (Canadian Association of Occupational Therapy, 2020; World Federation of Occupational Therapy, 2020). This critical interpretive synthesis (CIS) examines how the concept of oppression has been used in occupational literature, and whether more explicit attention to oppression might prove beneficial.

### The Concept of Oppression

Oppression is a concept that developed “in the shadows” of concepts such as equity and justice (Cudd, 2006, p. 4). Theorists and social scientists in both the global North and global South use the term oppression and some have argued that oppression should be one of the primary terms used to conceptualize injustice (Freire, 1970/2000; Young, 1990). In fields such as education and social work, anti-oppressive practice has an extensive history (e.g., Delpit, 1988; Dominelli, 1996; Freire, 1970/2000). Oppression always involves harm due to social group membership, always involves the corresponding benefit to a different group, is always systemic or structured within a society, and is always rooted historically (Cudd, 2006; David & Derthick, 2018; Freire, 1970/2000; Young, 1990). Whether the harm is intentional or unintentional, overt or covert, and unconscious or conscious (David & Derthick, 2018), if the situation involves these characteristics, it is oppression. Narrower foci such as prejudice (biased prejudgments) and discrimination (actions and inactions arising from prejudice) may point toward solutions (such as education or legislation), but without understanding of oppression, may not attend to root causes (Dovidio et al., 2010). Naming oppression necessitates attention to systematic relations of power implicating multiple interconnected social structures, such as media, education, legal systems, housing, politics, and the economy (Cudd, 2006; Freire, 1970/2000). Whether oppression operates through ideologies, laws, policies, institutional practices, or interpersonal interactions (often called “microaggressions”), there may be direct effects on occupations. Specifically, microaggressions are the innocuous everyday communications that convey negative messages of marginality to members of subordinated groups, resting on broader historical and contemporary social power relations (Sue et al., 2007).

Power is central to the operation of oppression (Harvey, 2010), maintained through force, ideologies, social policies, the threat of violence, and ways of thinking that become so dominant as to appear inevitable and uncontestable (Clifford, 2016; Collins, 2000). Power relations of domination and subordination are usually socially approved, backed by norms of status and authority (Harvey, 2010). Power is identified within specific systems of oppression, such as racism, ableism, and sexism, that lead to experiences of social marginalization, exploitation, powerlessness, violence, and/or cultural imperialism—the imposition of dominant worldviews that render one's own life invisible or inferior (Young, 1990). In situations of oppression, harmful inequities arise “systemically as the result of enduring concentrations of power and resources in the hands of some social groups at the expense of others” embodied “in concrete structural, institutional and organisational as well as psychological realities” (Clifford, 2016, p. 5).

The concept of intersectionality (Crenshaw, 1989) captures how simultaneous membership in multiple social groups—some oppressed, some privileged—shapes experiences of power and oppression in complex non-additive ways. Emerging from a rich heritage of advocacy and scholarship by Black women, intersectionality describes “how complexities of intersecting systems of power complicated everything in their lives” (Collins, 2015, p. 2350). For example, when White women in the global North were encouraged to stay home with children, most Black women were required to work outside their homes (Collins, 2015). Both are intersections of racism and sexism. Similarly, living with disability intersects with financial status to affect very different experiences and outcomes by social class (Stabile & Allin, 2012).

Oppression, by virtue of naming a moral wrong, invokes responsibility for action (Cudd, 2006). In his classic text on oppression, Freire (1970/2000) argues that the first step in considering action is conscious raising (conscientizaçao), a process in which experiences of oppression and their causes are identified. Oppression can then be understood “not as a closed world from which there is no exit, but as a limiting situation which [people] can transform” (Freire, 1970/2000, p. 49). This is an inherent part of *praxis* defined by Freire as “reflection and action upon the world in order to transform it” (Freire, 1970/2000, p. 51), transforming not just consciousness, but oppressive systems and social structures.

### Oppression and Occupational Therapy

Although theories of oppression come from beyond occupational therapy, related concepts have been considered within occupational therapy and occupational science (e.g., Gupta and Garber, 2017). Occupational scholars have examined structural forces that limit and demand occupational participation (Hammell, 2020). They have considered intersectionality (Angell, 2014), consciousness raising and occupational consciousness (Ramugondo, 2015), and the effects of power (Kiepek et al., 2019; Pyatak & Muccitelli, 2011). Scholars have encouraged therapists to seek justice, to name and address root causes of inequities, and to be aware of systemic influences (Farias et al., 2016; Gupta & Garber, 2017; Ramugondo, 2015; Silvestrini et al., 2019). Therapists have been challenged to consider the impact of oppression on occupational engagement (Angell, 2014; Ramugondo, 2015) and whether they perpetuate oppression with colleagues (Chacala et al., 2014) and clients (Kirsh et al., 2006). Occupational therapists have been asked to stand against oppression while promoting inclusion and equity (Carlsson, 2009). Most recently, literature in occupational therapy has begun to challenge the pervasiveness of dominant worldviews within the profession, those of White supremacy (Grenier, 2020), those of settler colonialism (e.g., Emery-Whittington & Te Maro, 2018; Fijal & Beagan, 2019; Gerlach et al., 2018; Gibson, 2020; Occupational Therapy Association of South Africa, 2020; White & Beagan, 2020), those of normative ableism (e.g., Gappmayer, 2021; Grenier, 2021), and those of class privilege (e.g., Hocking, 2019).

Yet, despite attention to ideas and concepts related to oppression, the term itself has been largely absent, at best implicit, in occupational therapy and science literature. An April 2021 search of the Combined Index of Nursing and Allied Health Literature (CINAHL) with oppression, occupational therapy, and occupational science as keywords returned only 11 citations, four of them from journals in the global South; all but one have never been cited or have been cited only once or twice, though two papers date back to 1996. Perhaps “oppression” has been too strong a term for the profession (Beagan, 2020)? The recent literature identified above indicates that the profession is attending to structured power relations. The concept of oppression, with its insistence on systemic causes, interrogation of domination and privilege, and identification of harm due to social identities, may add to this discourse and contribute to a needed plurality of ways to examine harm and opportunities for change (Guajardo Córdoba, 2020). Clarity about what constitutes oppression (ongoing benefit or harm to social groups, historically rooted, embedded in multiple interlocking social systems) may also help to avoid seeing every instance of discomfort or underrepresentation as a social justice concern (e.g., seeing the small number of men entering traditionally feminine jobs as discrimination; Beagan and Fredericks, 2018). Research on specific axes of oppression—racism, colonialism, sexism, ableism, classism, ageism—is valuable and necessary, yet there may also be an important place for the concept of “oppression” in the conceptual toolbox of occupational therapy.

This CIS examines how the concept of oppression, and terms naming axes of oppression, have been used in occupational literature. We explore whether explicit attention to oppression might contribute to understanding of injustice within occupational therapy. Attending to oppression at multiple levels in occupational therapy, scholarship, and teaching may benefit learners and practitioners who need to navigate constraints and opportunities of their practice settings and also the broader social power relations within which they are inevitably located.

### Methodology

CIS uses a critical perspective to create a synthesizing argument or theoretical proposal, grounded logically and plausibly in the data (Dixon-Woods et al., 2006). Beginning from a subjective idealist epistemological position (Barnett-Page & Thomas, 2009), researchers use a broad literature search strategy and tentative research question to find relevant literature. The process is iterative and recursive, with overlapping data collection and analysis, and may employ sampling strategies that maximize variation (Entwistle et al., 2012). In response to the emerging findings, non-linear processes of analysis and critique are selected and theoretical saturation may determine when the literature has been sufficiently examined (Dixon-Woods et al., 2006). Analytic processes are not prescribed but are to be critical, reflexive, dialogic, and creative as information is interpreted and themes are recognized (Dixon-Woods et al., 2006).

In keeping with critical traditions, as authors, we acknowledge that our positionality shapes our values and beliefs in ways that become inseparable from the research process. The first author is an experienced occupational therapist well-read in occupational justice, injustice, and rights, who nonetheless felt ill-prepared to engage with the realities of oppression in practice. Her commitment to social change, occasioned first by sustained engagement with the words and ideas of Indigenous peoples, pushed her to examine multiple approaches to critical thinking and acting. The second author teaches about oppression in occupational therapy. Both authors identify as settler, white, cisgender, currently middle-class, able-bodied women; the second author also identifies as a lesbian of lower class origins.

### Sampling

Unlike a scoping review, intended to survey existing literature comprehensively (Arksey & O’Malley, 2005), CIS proposes non-linear, purposeful sampling, not aspiring to representativeness so much as interpretive potential (Dixon-Woods et al., 2006). For this CIS, the databases CINAHL, PsycINFO, PubMed, and Scopus were searched (1998–2019) for occupational therapy/therapist(s) or occupational science and oppress* as keywords. Finding almost nothing, the search was run with more specific keywords sexism, racism, classism, ableism, disablism, ageism, heterosexism, and colonialism, to retrieve papers that might address oppression without using that language. Articles were retained if they were peer-reviewed and English language, resulting in 104 articles. After exclusion criteria were applied (eliminating articles that did not contain any of the keywords, those with no reference to occupational therapy or science, and published in journals outside those fields), 73 articles remained. Abstract and/or full-text review was used to determine the depth to which oppression was explored within individual articles. This reading informed the selection process within a sampling frame. To obtain the articles for inclusion, the articles were divided by subject (oppression, racism, sexism, ableism, heterosexism, classism, ageism, intersectionality) and time period (1998–2005, 2006–2012, and 2013–2019). One article was selected from each of these 24 subject/time groups (where possible; some categories contained no articles), with occasional additional articles added to enhance depth of analysis and diversity of literature included. The final selection of 25 articles was done by the first author in dialogue with the second.

### Data Analysis, Rigour, Limitations

The sample of 25 articles was read multiple times while examining their conceptual framing. A full-text review of three articles was used to shape the data collection process, which was then expanded to include ideas from oppression theories. For each article, a brief interpretive summary was created to encapsulate how naming oppression contributed to the article. Quotes were extracted that illustrate the choice of terms used to clarify the experience of oppression, the actions people take following experiences of oppression, how power is addressed, and how occupational therapy and occupational justice terms are used by the authors. Throughout the data collection process, reflective comments were recorded in a journal and reflexive, critical discussion between authors supported the iterative analysis.

Considerations of saturation were left until the initial data had been fully examined so that emerging insights could influence the process (Dixon-Woods et al., 2006). When assessing saturation, three additional peer-reviewed, English language articles written by authors not included in any of the initial 25 studies were chosen (for a total of 28 included articles). The information in these articles did not challenge the analysis arising from the initial set of articles nor require the creation of additional categories to express key oppression-related content; it was determined that theoretical saturation had been attained.

Although efforts to ensure rigour were undertaken, there are limitations to this research. First, this CIS focuses on work published in English, peer-reviewed journals. Other sources (i.e., conference proceedings) were briefly reviewed, but not included. Second, the number of similar terms available in English, as well as any stigma associated with particular terms, may have affected results. For example, scholars sometimes use culture instead of race, or class inequity instead of classism and this study was limited to sources using the “ism” terms. Third, database design limits the articles that can be accessed. Databases are biased toward Western/Northern journals and may prioritize quantitative or positivistic constructs over social concepts (Patin et al., 2020). Finally, time and resource constraints may have limited the creative nature of the CIS processes. Although all of these factors limit the analysis presented, it is also true that occupational therapy and occupational science literature that directly addresses oppression is a finite volume. The analysis presented captures the gestalt or overall character of that body of literature, even if it misses some individual pieces.

## Findings

The articles included in this CIS use qualitative, quantitative, mixed methods, literature reviews, and theoretical analysis. The studies include people who live with physical and mental health challenges in nine countries; children, adults, and senior citizens; occupational therapy students and occupational therapists; and clinical and non-clinical situations. The themes identified indicate a strong fit with the concept of oppression: Everyday doing is hindered by microaggressions, invisibility, and violence; constraints on occupations are rooted in structural and institutional power relations, as well as dominant ideologies; responding to or resisting oppression always involves agency within constrained “choices” and is often stressful, yet resistance occurs on multiple levels; occupational therapy is not immune from perpetuating oppression and suggestions for change are plentiful. Each theme refers not to a discrete group of articles, but cross-cutting ideas found in the sample.

### Oppression and Everyday Doing

As Ramugondo (2015) has stated, “everyday doing intersects with oppression” (p. 495). Whether they were examining ageism, ableism, classism, colonialism, heterosexism, racism, sexism, or intersectional experiences, the authors included in this CIS described numerous ways everyday occupations were altered by oppression. In a study of racism, one participant stated, “It impacts everything I do, everything I say, everything I wear, everything I eat, people I talk to . . . most life activities. It's so pervasive” (Beagan & Etowa, 2009, p. 288). Forms of oppression were shown to affect everyday occupations from riding in taxis (Bergan-Gander & von Kürthy, 2006), to accessing health care (Gerlach, 2008), from interactions with professional colleagues (Chacala et al., 2014) to farming practices (Simaan, 2017). Authors described experiences that involve exploitation in work and hospital environments (Bailliard, 2013; Crabtree, 2005; Howarth & Jones, 1999), marginalization in educational, health, and community settings (Angell, 2014; Joubert, 2010; Trentham & Neysmith, 2018) and fear of violence (Gerlach, 2008; Wicks, 2001).

Many of the incidents described can be seen as microaggressions (Sue et al., 2007), which may shift the meaning of occupations. For example, for racialized or disabled children, being teased mercilessly can take the joy out of schoolyard games (Angell, 2014). For Latino migrants, enduring the gaze of strangers who “look at you as if you were a cockroach” (Bailliard, 2013, p. 351) can infuse work, driving, or any daily occupation with lack of belonging. Similarly, for racialized women, “being ignored, being followed, being treated disrespectfully, not being taken seriously” (Beagan & Etowa, 2009, p. 288), can obliterate any pleasure experienced while shopping. Experiences of health care can be negatively affected by practitioner attitudes such as when a racialized client is treated patronizingly, assumed not to speak English (Kirsh et al., 2006).

In addition to microaggressions, the papers examined here explored how occupations may be affected by invisibility, internalization of oppression, and fear of violence. In one study, a participant shared that due to ageism, “our lives as citizens are rendered invisible and we battle to find the language, let alone define strategies, to delineate alternatives” (Trentham & Neysmith, 2018, p. 181). When a stigmatized aspect of identity is highlighted, other aspects can be rendered invisible, constraining occupational opportunities: “They looked at my disability, not my brain” (Joubert, 2010, p. 24). The authors also examined how racism, sexism, heterosexism, ageism, and ableism may be internalized, leading people to “choose” to constrain or alter their own occupations. For example, internalized ageism can lead to efforts to appear younger (Trentham & Neysmith, 2018), and internalized gender roles may “become encoded as individual self-expression and individual choice” (Beagan & Fredericks, 2018, p. 141). Most overtly, perhaps, violence and fear of violence may circumscribe occupations. For example, Latino migrants afraid of police hostility avoided travel for groceries, relying on canned goods that reduced gustatory pleasure and cultural continuity (Bailliard, 2013).

### Effects of Structures and Power

With all forms of oppression, harm may be experienced at an individual level, but the causes of such harms reside in social and institutional structures and systematic power relations. Attention to structures that result in oppressive harm is apparent in this group of articles, as authors identify harms rooted historically and contemporarily in legislation such as the Indian Act in Canada (Gerlach, 2008), the REAL ID Act in the United States (Bailliard, 2013), and the British mandate in Palestine (Simaan, 2017). Such “macro” level structures influence “meso” levels, shaping policy and organizations. Within the selected studies, the authors examine such institutional processes as welfare policies (Boland & Cunningham, 2019), paternalistic organizational practices with older mental health consumers (Fortune et al., 2007), and funding mechanisms that mandate individual responsibility for “productive aging” (Laliberte Rudman & Molke, 2009).

The selected articles include examples of relationships between structures and occupation, showing how oppression can lead to unwanted occupation when resources and opportunities are distributed in ways that benefit some people instead of others. For example, limited work options available to undocumented workers lead to exploitation: “If you don’t want it, another will come who does . . . you submit” (Bailliard, 2013, p. 350). Policies can create marginalizing conditions by limiting who can live in certain neighbourhoods (Pyatak & Muccitelli, 2011) or work in certain locations (Simaan, 2017). Such structures and policies create powerlessness, wherein people have insufficient influence to implement their occupational preferences (Boland & Cunningham, 2019; Young, 1990).

Power operates not only through structures and material resources but also through the circulation of dominant ideas as “normal” and “common sense” (Collins, 2000). The selected articles examine numerous instances where dominant beliefs constrain occupation, including messages about which age groups should be productive (Laliberte Rudman & Molke, 2009), which people are presumed independent (Chacala et al., 2014), and whose disruptive behavior is dismissed as natural, such as “boys will be boys” (Angell, 2014, p. 112). Authors draw connections between ideas of “normal” gender or class expectations and job choices or hiring practices; those employment patterns then recursively reproduce gender and class oppression (Beagan & Fredericks, 2018; Hammell, 2013).

### Responding and Resisting

The authors of the selected articles also attend to how people make difficult “choices” to respond to and/or resist oppression. Participants might not respond to protect themselves against harm, might accommodate oppressive circumstances to ease daily living, and/or might act to resist or reduce oppression. Attending to occupational responses and resistance recognizes the agency of people in situations of oppression, yet these can all still be considered constrained choices (Angell, 2014; Simaan, 2017).

The constraints imposed by oppressive structures were evident when authors explored people's “choice” not to respond to oppression. In one study, parents taught children to avoid reacting to racism: “Let it roll off because you’re going to have it in your everyday life, eventually, all the time” (Beagan & Etowa, 2009, p. 291). For Latino migrants, eliminating activities reduced opportunities to encounter harm (Bailliard, 2013), while for homeless people refusing participation in activities organized for them “demonstrated agency and identity against the oppressive rules” (Boland & Cunningham, 2019, p. 312). Some study participants strived to make situations more tolerable by making constrained choices such as “passing” as members of a non-stigmatized group such as heterosexuals (Bergan-Gander & von Kürthy, 2006) or non-seniors (Trentham & Neysmith, 2018), or by reappropriating racist terms (Pyatak & Muccitelli, 2011). Commonly, occupational engagement involved discerning when to take action: “You forgive fast and move on. Sometimes I call out the racism, sometimes I sit back and hope my allies will” (Emery-Whittington & Te Maro, 2018, p. 18).

When resisting oppression, raising consciousness proves a frequent starting point (Emery-Whittington & Te Maro, 2018; Joubert, 2010; Pyatak & Muccitelli, 2011; Townsend et al., 2000). Ramugondo (2015) argues that occupational consciousness is essential to recognize the influence of dominant practices sustained by and through everyday occupations. Actions to resist oppression could be fleeting, such as the deliberate behaviour of Black boys to disrupt power dynamics in the classroom (Angell, 2014), or steadfast and obdurate, such as relentlessly replanting trees on occupied lands “until the aggressors give up” (Simaan, 2017, p. 518). Resistance could be directed at immediate situations or aimed at improving broader social and policy contexts, such as seniors producing advocacy materials on ageism, support services, and funding structures (Trentham & Neysmith, 2018). Resistance occupations may take the form of protest and activism (Nelson, 2007), or producing art: “Rap music has developed as a means of resistance by which Black Americans can call attention to the political, social, and economic conditions faced by their communities and the postcolonial racist practices that serve to sustain these conditions” (Pyatak & Muccitelli, 2011, p. 51). Deciding when, whether, and how to respond to oppression, in the context of ongoing constraints, can be emotionally exhausting—as noted by one participant resisting racism, “I went and challenged them . . . that's very, very stressful to have to go through that” (Beagan & Etowa, 2009, p. 289).

### Oppression Within Occupational Therapy

As an institution, the profession of occupational therapy has perpetuated oppressive harms. Articles in this CIS document ableism, ageism, colonialism, racism, and sexism within occupational therapy examining experiences of clients, therapists, and students. Within these articles, a therapist is dehumanized and called a fire hazard (Chacala et al., 2014, p. 112), clients describe feeling invisible (Kirsh et al., 2006), bias toward male clients is found in an assessment (Jong et al., 2012), and therapists realize the discordance between the occupations they anticipate for themselves in older adulthood and the occupations they expect for clients (Alden & Toth-Cohen, 2015) (see also Table 1). The authors examine how power dynamics such as those present in ableism and colonialism shaped the profession's development and continue to influence interactions (Joubert, 2010; Nelson, 2007). Theoretical frameworks employed include critical discourse analysis (Laliberte Rudman & Molke, 2009), Black feminist theory (Angell, 2014), and decolonial theory (Emery-Whittington & Te Maro, 2018), demonstrating several authors’ commitment to incorporate power as a central part of their theoretical lens.

**Table 1.** Examples of Oppression Experienced in Occupational Therapy Settings

System of Oppression	Example of Oppression
Ableism	A blind occupational therapist recounts: “I’ve been called a fire hazard, I’ve been stopped going on courses. Just horrific, some of the comments.” (Chacala et al., 2014)
Ageism	Through workshops, occupational therapists “realized they had different expectations for themselves than for their clients” (Alden and Toth-Cohen, 2015, p. 11).
Colonialism	Maori occupational therapists in a keynote address tell colleagues “We bear witness to resounding silence when we question assumptions and put up with regular assessment of our ‘Maori-ness’ on an imaginary scale of authenticity” (Emery-Whittington and Te Maro, 2018, p. 13).
Racism	Clients described “A sense of ‘invisibility’, of being excluded from the occupational therapy process” (Kirsh et al., 2006, p. 310).
Sexism	An assessment tool with a bias toward male occupations may contribute to a gender bias in access to services (Jong et al., 2012).

Calls for change, proposed by the authors of the selected studies, insist that therapists create a critical, reflexive discipline (Angell, 2014), ask critical questions (Beagan & Etowa, 2009; Townsend et al., 2000), and develop “networks of critical friends and safe reflective spaces” (Nicholls & Elliot, 2019, p. 10). Authors advocate that therapists interrogate their assumptions about who should participate in particular occupations (Laliberte Rudman & Molke, 2009), and question how they may be interpreting structural inequities as individual client failings (Beagan & Etowa, 2009). Such critical self-questioning may necessitate “deep and sometimes painful interrogation” (Joubert, 2010, p. 21; see also Nicholls & Elliot, 2019). On the other hand, as Steed (2014) documents, even extended educational interventions may not shift entrenched racist beliefs of occupational therapy students. Alden and Toth-Cohen (2015) found similar results with education to reduce therapist ageism and “elderspeak.”

Scholars’ recommendations for occupational therapists reflect their desire for change and include creating space for clients to discuss the impact of oppression, and assuming client occupations are affected by ageism (Trentham & Neysmith, 2018), racism (Beagan & Etowa, 2009), and heterosexism (Bergan-Gander & von Kürthy, 2006). At the community level, therapists/activists (Pyatak & Muccitelli, 2011) are encouraged to advocate for occupational rights and justice (Boland & Cunningham, 2019). Within the profession, therapists are urged to promote respectful interactions by challenging oppressive assumptions (Chacala et al., 2014; Howarth & Jones, 1999; Townsend et al., 2000), ensuring they are not reinforcing stereotypes (Alden & Toth-Cohen, 2015; Beagan & Etowa, 2009; Steed, 2014), and creating student experiences that disrupt social norms (Stewart et al., 2005). Therapists and scholars are pressed to address oppression embedded in policy (Howarth & Jones, 1999), data collection (Beagan & Fredericks, 2018; Stewart et al., 2005), written texts (Laliberte Rudman & Molke, 2009), assessment tools (Jong et al., 2012), evaluation frameworks (Fortune et al., 2007), and dominant professional epistemologies (Gerlach, 2008; Nicholls & Elliot, 2019). Compared to the wide-ranging scope of recommendations, the selected articles describing actual changes denote relatively small initiatives such as creating a research group in a mental health day program (Townsend et al., 2000), advocacy work with senior citizens (Trentham & Neysmith, 2018), and implementing education programs for current/future therapists (Alden & Toth-Cohen, 2015; Steed, 2014).

## Discussion

Through this CIS, it is clear that some scholars in occupational therapy and science are publishing work that is congruent with the concept of oppression, in that it examines harms and benefits at the level of social groups, it examines harms that are systemic and structural, occurring at micro, meso, and macro levels, and it examines power relations as causal. Employing critical reflexivity, power relations are identified not just “out there” but also within the profession, in encounters with clients, colleagues, and students. Most scholars appear to be using language aligned with specific axes of oppression: racism, ageism, colonialism, and so on. The findings demonstrate that our profession is examining multiple forms of oppression across practice areas. The term oppression itself, however, rarely arises in occupational therapy.

The notion of oppression insists on structural, systemic power analyses while “refusing to hide the rawness of what power relations of racism, sexism, class exploitation and heterosexism does to people on the bottom” (Collins, 2015, p. 2350). This focus may help occupational therapists to avoid individualist explanations that pathologize inequities (Gerlach et al., 2018). Naming oppression also promotes intersectional analysis (Rosenthal, 2016) and disrupts the idea that the *status quo* is natural rather than structural in origin (Neff et al., 2019). Such approaches move toward “structural competency,” the work of identifying structural determinants that influence health and well-being (Metzl & Hansen, 2014). Structural competency attempts to bridge individual and institutional causes of poor health, encouraging interdisciplinary and community collaboration and promoting long-term systemic solutions (Metzl & Hansen, 2014). Increasing the structural competency of the profession may encourage occupational therapists to identify social structures that constrain opportunity (Hammell, 2015) and to be unsatisfied with naming (individual) social determinants of health without examining their structural causes (Neff et al., 2019).

A plurality of approaches to injustice, which includes the concept of oppression, can disrupt universalist and homogeneous ideas that have been associated with a Western understanding of justice (Guajardo Córdoba, 2020). Understanding oppression brings an unwavering focus on root causes and harmful outcomes to discourse related to justice. It is not the only term to address root causes, but the sustained attention inherent in oppression suggests this concept should be considered for use when foci include social structures and power. Both the term inequity and critical theory have been criticized for avoiding attention to social structures and causes (Arcaya et al., 2015; Sayer, 2009). Occupational justice (Townsend & Wilcock, 2004) has also been criticized for not adequately addressing causes that determine social inequalities (Guajardo Córdoba, 2020). With oppression, the idea of an “oppressor” insists upon examination of the people and structures that contribute to and benefit from harm (Clifford, 2016). The concept of oppression need not replace the politicized language of racism, heterosexism, or other terms connected to social justice, but it may provide another useful tool for critical scholars to support occupational therapy's work toward equity, justice, and social change.

The concept of oppression can also add strength to critiques of depoliticized language, such as “cultural difference” rather than racism, “gender roles” rather than sexism, and “age-related experiences” rather than ageism. Such misdirections not only fail to name root causes of inequities, but they also fail to point to the benefits that accrue to dominant social groups, ultimately misidentifying avenues for change (Beagan, 2015; Stewart, 2000). Similarly, additional attention to structured power relations of domination and privilege may help scholarship move away from what may be a more politically palatable focus on “diversity” or “disadvantage” (Macedo, 2014; Stewart, 2000). At the same time, a broader conceptual understanding of oppression may help the profession to identify harms that occur across social groups (e.g., invisibility, internalization, microaggressions) and promote the recognition of intersectionality (the non-additive implications of membership in multiple social groups). The clarity of the concept of oppression may help reduce the silencing of harms that can happen with depoliticized language while focusing attention on the relationships between harm, structures, and everyday occupations.

Oppression by definition identifies moral wrongs and these situations of injustice require action (Cudd, 2006). Oppression, long used in the global South in emancipatory education contexts (Freire, 1970/2000), is a concept that demands praxis, the wedding of analysis and action. This requires unity in collective struggle, as equals; it requires organization around the causes of oppression, demanding critical knowledge of sociohistorical context; it requires taking leadership from the critical analyses of people living with oppression (Freire, 1970/2000). Attention to oppression may extend a moral imperative for praxis, supporting an emancipatory agenda (Farias & Laliberte Rudman, 2016; Hocking & Whiteford, 2012). Developing the skills for critical analysis is a necessary first step in praxis and can be supported by growing consciousness and structural competency. An oppression framework suggests that equal partnerships and collective action are essential next steps.

Praxis demands that attention to analysis be balanced with energy and resources directed at change. Although taking time to understand the complex concepts and contexts can reduce the potential for further harm (Thibeault, 2013), the relatively small changes described in the selected articles suggests that there is room for greater focus on action and change. At an individual level, a robust notion of client-centred that fully integrates critical reflexivity can reduce oppression in everyday practice. In addition, therapists can take time to listen to others who name oppressions, identify structures and procedures that limit occupation, and direct effort toward change as part of enabling occupation. At a professional level, modifications to policies that guide energy and resources may change who is able to access occupational therapy and who feels welcome and valued (Kendi, 2019). Greater attention to structural and systemic power in research and education settings may promote structural competency and strategic action. Naming oppression when appropriate (e.g., ageism instead of “age related experiences”) might support this process by challenging hesitancy to call things what they are (using politicized language) and promote contextual thinking. Collective next steps, particularly interdisciplinary ones, can work toward wider system change. The occupational therapy profession may also examine further opportunities to reduce oppression based on community priorities through approaches such as Social OT (Malfitano & Lopes, 2018) and Occupation-Based Community Development (Galvaan, 2021).

Future research may offer a greater understanding of the relationships between the themes that emerged in this study, occupational performance, and constrained choices. When beginning research related to justice and equity, information search strategies that focus uniquely on terms such as justice, equity, diversity, and inclusion without oppression(s) may miss the expertise of people with lived experience who choose this language, and people, particularly in the global South, who prioritize a focus on context over (assumed) universality. Further research might support both policy changes to reduce oppression and lead to more nuanced knowledge about the meaning of occupations when limited or demanded by unjust constraints. There is more research supporting theme one (oppression and everyday doing), thus increased examination of power, social structures, and resistance in relation to occupation is encouraged. For example, further exploration of resistance within health and social care systems could examine how clients, therapists, and professional groups resist within healthcare hierarchies.

Attention to these themes in practice, teaching, and future research can help demonstrate that occupational therapy is “not neutral and has a theoretical and scientific praxis committed to the oppressed other” (Guajardo, 2017, p. xvi). Including the concept of oppression within the plurality of social discourse can enhance occupational therapy, emphasizing root causes, power structures, and strategic, critically reflexive action to contribute to meaningful social change.

## Conclusion

This CIS suggests that scholars in occupational science and occupation therapy are engaging in work that addresses specific axes of oppression, such as ableism, racism, and colonialism. This work interrogates the impacts of oppression on occupations, examines the roots of oppressions in structured power relations, illustrates the constrained choices and agency of people who decide when and how to respond and resist oppressions, and reflexively analyzes the operation of oppressive power relations within the profession. Yet, oppression itself remains an under-used concept in the lexicon of occupational therapy and may prove a useful addition, helping to extend the focus on structures of power, intersectionality, and praxis.

## Key Messages

- Oppression and systems of oppression (e.g., racism, ageism) identify systemic harm to social groups and focus attention on root causes of injustice, yet are underexamined in occupational therapy.
- The concept of oppression can contribute to a needed plurality of ways to examine social harm and opportunities for change within the profession of occupational therapy.
- Oppression as a guiding concept moves analyses toward structural causes of injustice, avoiding individualist understandings that blame those who are oppressed.
